# Training gaps and needs of pre-hospital emergency staff in Chengdu: a city-wide cross-sectional study

**DOI:** 10.3389/fmed.2026.1806022

**Published:** 2026-04-30

**Authors:** Chengzhi Hu, Xue Xue, Haixia Li, Baoli Xu, Peng Jiang, Yi Pan, Minghui Ye

**Affiliations:** 1 Department of Training, Chengdu Emergency Medical Services Command Center, Chengdu, Sichuan, China; 2 Department of Emergency, West China School of Medicine, Sichuan University, Sichuan University Affiliated Chengdu Second People’s Hospital, Chengdu Second People’s Hospital, Chengdu, Sichuan, China; 3 Department of Emergency, Chengdu Women’s and Children’s Central Hospital, Chengdu, Sichuan, China; 4 Department of Emergency, Sichuan Lansheng Neurological Hospital, Chengdu, Sichuan, China

**Keywords:** pre-hospital emergency care, pre-hospital emergency personnel, targeted training, training needs, training status

## Abstract

**Objective:**

To understand the basic situation, training status, and training needs of pre-hospital emergency personnel in Chengdu, and to provide a scientific basis for developing standardized and unified pre-hospital training content and methods in Chengdu, with the aim of improving the quality of the pre-hospital emergency talent pool.

**Methods:**

An anonymous questionnaire survey was conducted. Questionnaires were distributed to 196 120-network hospitals in Chengdu, covering basic information, training status, and training needs. Data analysis was performed using Excel and SPSS 26.0.

**Results:**

A total of 4,209 pre-hospital emergency personnel participated in the survey. The main way of acquiring first aid knowledge was in-person training organized by their institutions (93.68%, 3,943 person-times). The main training content attended was practical operations (94.65%, 3,984 person-times). Most personnel participated in first aid-related training less than twice per year (57.23%, 2,409 persons). The most significant factor affecting their participation in training was concern about whether training quality and practical relevance of content could be guaranteed (56.02%, 2,358 person-times). The training method most expected by pre-hospital emergency personnel was practical operation (84.77%, 3,568 person-times), and the most expected training content was environmental injuries (77.17%, 3,248 person-times). There was no significant difference in the ways of acquiring medical first aid knowledge among emergency personnel from hospitals of different levels (*P* > 0.05). There was no statistically significant difference in training method preferences among pre-hospital emergency personnel from hospitals of different levels (*P* > 0.05). There was a statistically significant difference in training content preferences among pre-hospital emergency personnel from hospitals of different levels (*P* < 0.05).

**Conclusion:**

The current first aid training in Chengdu cannot meet the training needs of pre-hospital emergency personnel. Training institutions should fully understand the characteristics of participants and conduct stratified training for pre-hospital emergency personnel from hospitals of different levels, implementing practice-oriented and highly practical training to improve the first aid capabilities of pre-hospital emergency personnel and promote the sustainable development of emergency medicine.

## 1 Introduction

Pre-hospital emergency care is the forefront of the emergency medical system, referring to the emergency treatment, transportation, and en-route care provided at the scene for various life-threatening acute and critical conditions (1). Affected by factors such as population, transportation, distribution of medical institutions, and patient conditions, some patients cannot receive timely treatment at hospitals, making pre-hospital emergency care particularly important (2). Pre-hospital emergency personnel need to adopt the most effective emergency diagnosis and treatment techniques in the shortest possible time to save the lives of various acute and critically ill or injured patients, requiring strong psychological resilience and technical adaptability. The United States not only has a complete emergency knowledge and skills training system but also established standard emergency skills assessment criteria; France’s pre-hospital emergency personnel have early clinical exposure, abundant opportunities to participate in emergency practice, and long training cycles—even drivers are required to undergo emergency theory training and operational exercises—resulting in generally complete training models and high levels of knowledge and skills; Germany’s pre-hospital emergency care mainly relies on specially trained physicians holding emergency qualification certificates (3). In China, pre-hospital emergency personnel must pass pre-job training and assessment before taking up their posts, but currently there is no unified pre-job training content or method, and the assessment and evaluation system also lacks uniform standards (4–6). The emergency capabilities of pre-hospital emergency personnel vary significantly across different hospitals, and issues such as heavy workloads, poor compensation, and high turnover rates among emergency personnel in China have gradually become prominent, becoming bottlenecks constraining the development of pre-hospital emergency care. As the main base for emergency training in Chengdu, the Chengdu Emergency Medical Command Center bears the important responsibility of improving pre-hospital emergency personnel’s knowledge and skills, enabling patients to receive rapid, effective, and high-quality pre-hospital treatment, and reducing disability and mortality rates (7, 8). This survey aims to investigate pre-hospital emergency personnel throughout the city to provide a basis for developing standardized and unified training content and methods, with the expectation of improving the emergency capabilities of pre-hospital emergency personnel citywide.

## 2 Subjects and methods

### 2.1 Participants

From June 2024 to September 2024, a survey was conducted on pre-hospital emergency personnel from 196 120-network hospitals under the Chengdu Emergency Medical Command Center. Inclusion criteria: (1) Engaged in pre-hospital emergency-related work; (2) Voluntary participation in the survey. This study has been approved by the Medical Ethics Committee of Sichuan Lansheng Brain Hospital, approval number: S-20260311.

### 2.2 Survey procedure

First, Cronbach’s alpha coefficient was used to test the internal consistency of the questionnaire. The obtained Cronbach’s α value was 0.845, indicating that the questionnaire has good internal consistency and high reliability, capable of measuring research variables in a stable and reliable manner. Second, the structural validity of the questionnaire was tested through factor analysis. The KMO value was 0.892, significantly higher than 0.8, indicating that the data were suitable for factor analysis. In addition, the *p*-value of Bartlett’s test of sphericity was less than 0.05, further demonstrating that the data structure was appropriate for factor analysis. Based on the reliability and validity tests, the questionnaire items and option settings were revised to ensure the stability, reliability, and validity of the questionnaire, ultimately forming the official version of the Pre-hospital Emergency Training Needs Survey Questionnaire.

Two professional training staff then created an electronic version of the questionnaire using the Wenjuanxing platform. A unique QR code was generated and sent to every network hospital.

Each facility nominated one doctor and one nurse to coordinate the survey; these coordinators received a short briefing on the study’s purpose and completion guidelines. Responses were anonymous and one-time-only; the questionnaire could be submitted only after every item had been answered.

### 2.3 Statistical analysis

Data was processed with Excel and SPSS 26.0. Single-response items are reported as rates or proportions; multiple-response items are presented as counts and percentages. Group comparisons were performed with the χ^2^-test, and *P* < 0.05 was considered statistically significant.

## 3 Results

### 3.1 Baseline characteristics of pre-hospital emergency personnel

Between June and September 2024, the electronic questionnaire was sent to every pre-hospital professional affiliated with the 196 hospitals that form the Chengdu “120” network. In the end, 4,209 usable replies were received, a response rate that makes this the largest city-wide profile of its kind in western China. When the replies are broken down by institutional level, almost half of the respondents (2,024; 48.4%) work in tertiary-grade-A hospitals, the regional heavy-hitters that receive the most complex referrals—while a further 719 (17.2%) are based in tertiary-grade-B centers. Staff from secondary-grade-A, secondary-grade-B and sub-secondary hospitals contribute 383 (9.2%), 296 (7.0%), and 787 (18.8%), respectively, so the sample faithfully mirrors the stepped care structure that Chengdu residents meet in everyday life.

Educational attainment is heavily skewed toward the bachelor level: 2,673 respondents (63.5%) hold a first degree and another 1,233 (29.3%) have only junior-college or technical-school qualifications, meaning that 92.8% of the workforce possesses a bachelor’s degree or less. At the opposite extreme, only 27 individuals (0.6%) have doctorates and 276 (6.6%) master’s degrees, proportions that are markedly lower than those found among in-hospital specialists in the same city.

Experience is also polarized: 1,164 staff (27.7%) have been on the road for 5 years or fewer, 1,028 (24.4%) for 6–10 years, 1,357 (32.2%) for 11–15 years, and 660 (15.7%) for more than fifteen years. Consequently, just over half (52.1%) of the workforce could still be considered “early- to mid-career.” Professional titles follow the same pyramid: 1,367 respondents (32.5%) remain at junior rank, 1,676 (39.8%) have reached intermediate level, while only 331 (7.9%) have attained senior status and a mere handful are chief grade. Taken together, these figures portray a young, bachelor-level workforce that is unevenly distributed across the city’s three-tier hospital system and that has limited representation at the most senior academic or professional levels (Table 1).

**TABLE 1:** Basic information of 4,209 pre-hospital emergency medical personnel.

Item	Education				Years of work experience				Professional title		
	Doctorate	Master	Bachelor	Below bachelor	0-5 Y	5–10 Y	10–15 Y	<15 Y	Senior	Intermediate	Junior
No	27	276	2,673	1,233	1,164	1,028	1,357	660	331	1,676	1,367
Proportion (%)	0.64	6.56	63.51	29.29	27.66	24.42	32.24	15.68	7.86	39.82	32.48

#### 3.2 Current training exposure

Currently, the main way for pre-hospital emergency personnel in Chengdu to acquire first aid knowledge is participating in in-person training organized by their own institutions (93.68%, 3,943 person-times), followed by online training (86.20%, 3,628 person-times). Other important channels include participating in off-site training (74.27%, 3,126 person-times), reviewing literature and materials (73.94%, 3,112 person-times), attending academic lectures by medical associations (71.35%, 3,003 person-times), and watching popular science short videos (60.09%, 2,529 person-times).

The main training contents attended include professional theory (91.89%, 3,868 person-times) and firsthand practice (94.65%, 3,984 person-times). Other content areas covered include modern technologies (70.92%, 2,985 person-times), thinking methods (61.32%, 2,581 person-times), and research methods (32.17%, 1,354 person-times). Notably, less than one-third of personnel receive any training in research methods, indicating a significant gap in research literacy.

Most personnel participate in first aid-related training less than twice per year (57.23%, 2,409 persons), with 31.03% attending once yearly and 26.20% attending twice yearly. Only 19.86% attend three times per year, 15.02% four times, and merely 7.86% attend five times or more annually. This infrequent training pattern raises concerns about skill retention and currency in a high-stakes field (Table 2).

**TABLE 2:** Training status of 4,209 pre-hospital emergency medical personnel.

Item	No	Person-time ratio (%)
Primary source of knowledge acquisition		
In-person training organized by this institution	3,943	93.68
Online training	3,628	86.20
Attending academic lectures by medical associations	3,003	71.35
Participating in off-site training	3,126	74.27
Popular science short videos	2,529	60.09
Reviewing literature and materials	3,112	73.94
Training content attended		
Professional theory	3,868	91.89
Firsthand practice	3,984	94.65
Modern technologies	2,985	70.92
Thinking methods	2,581	61.32
Research methods	1,354	32.17
Frequency of emergency training attended		
1 time/year	1,306	31.03
2 times/year	1,103	26.20
3 times/year	837	19.86
4 times/year	601	15.02
5 times/year or more	331	7.86

There was no significant difference in the overall ways of acquiring medical first aid knowledge among emergency personnel from hospitals of different levels (*P* > 0.05). However, important nuances emerge when examining specific channels: emergency personnel from Tertiary A hospitals had the highest proportions of acquiring first aid knowledge through reviewing literature and materials (50.16%) and attending in-person academic lectures (50.18%), while emergency personnel from below-Secondary level hospitals relied more on off-site training (19.67%) and online training (18.27%). This pattern suggests that staff at higher-tier hospitals have greater access to academic resources and professional networks, whereas their grassroots counterparts depend more on centralized or digital training opportunities (Table 3).

**TABLE 3:** Ways of acquiring medical first aid knowledge in hospitals of different levels.

Item	Tertiary A	Tertiary B	Secondary A	Secondary B	Below Secondary	χ ^2^	*P*
Popular science short videos	1,210	458	249	164	448		
Reviewing literature and materials	1,561	548	293	183	527		
Off-site training	1,484	527	315	185	615	16.434	0.689
In-person training organized by this institution	1,927	683	379	239	715		
Online training	1,777	628	342	218	663		
Academic lectures by medical associations	1,507	518	293	173	512		

#### 3.3 Gaps identified

When respondents were invited to diagnose their own deficits, “professional operational skills” topped the list: 2,529 individuals (60.09%) ticked it as a weak area, followed by “clinical reasoning” (2,432; 57.78%), “professional theoretical knowledge” (2,041; 48.49%), “clinical experience” (1,741; 41.36%), “department management capabilities” (1,683; 39.99%), “communication ability” (1,592; 37.82%), and “research capability” (1,524; 36.21%). The hierarchy is consistent across hospital grades and mirrors the sequence of tasks a crew must perform at the roadside—skills first, cognition second, theory third.

Little wonder, then, that 3,599 respondents (85.51%) answered “yes” to the blunt question “Do you need further training?,” whereas only 610 (14.60%) felt they could safely stop learning. Asked what keeps them away from existing programs, the largest single deterrent—chosen by 2,358 (56.02%)—is “doubts about training quality or practical relevance of content,” a complaint that encompasses everything from outdated protocols to instructors who have not worked in the field for years. Logistical barriers are also powerful: 2,297 staff (54.57%) cannot go because “no replacement is arranged for duties,” 1,836 (43.52%) cite family-related issues, 1,818 (43.19%) mention high training costs, and 553 (13.13%) say their unit simply refuses to arrange coverage. Thus, the gap is simultaneously motivational and structural: crews want training that is credibly useful and compatibly scheduled, but their departments cannot or will not provide coverage (Table 4).

**TABLE 4:** Main problems reported by 4,209 pre-hospital emergency personnel.

Item	No	Person-time ratio (%)
Weak areas		
Professional theoretical knowledge	2,041	48.49
Professional operational skills	2,529	60.09
Clinical reasoning	2,432	57.78
Communication ability	1,592	36.82
Research capability	1,524	36.21
Clinical experience	1,741	41.36
Department management	1,683	39.99
Is further training needed?		
Yes	3,599	85.51
No	610	14.60
Main concerns about attending training		
Unit does not arrange coverage	553	13.13
No replacement of staff for duties	2,297	54.57
Family-related issues	1,836	43.52
Quality of training / practical relevance of content	2,358	56.02
High training costs	1,818	43.19

The pattern of self-identified weak areas varies significantly by years of work experience (χ^2^ = 203.797, *P* < 0.001). Staff with 0–5 years of experience most commonly identified clinical experience (60.4%) and communication skills (39.3%) as weaknesses, reflecting their newcomer status. Those with 5–10 years prioritized professional theoretical knowledge (49.1%) and operational skills (59.6%), suggesting they are consolidating their technical foundation. Mid-career personnel (10–15 years) showed the highest concern about clinical thinking (61.5%) and department management (44.1%), indicating growing administrative responsibilities. Senior staff (>15 years) were less likely to identify weaknesses overall, though operational skills remained a concern for 57.7%. Notably, research capabilities were consistently identified as weak across all experience groups, ranging from 32.3% in the most junior staff to 36.2% in the most senior, underscoring a systemic neglect of research training (Table 5).

**TABLE 5:** Differences in weak links among different years of work experience.

Item	0–5 years	5–10 years	10–15 years	15 above years	χ ^2^	*p*
Professional theoretical knowledge	603	505	638	295	203.797	0.000
Professional operational skills	734	613	801	381		
Clinical thinking	605	595	834	398		
Clinical experience	703	425	449	164		
Department management capabilities	394	415	598	276		
Communication skills	458	423	506	205		
Research capabilities	376	398	511	239		

### 3.4 Training preferences

#### 3.4.1 Preferred formats vary by hospital level

Offered six instructional formats and allowed to choose any number, respondents produced a clear ranking. Firsthand practice dominates: 3,568 (84.77%) want more manikin-based drills, simulation scenarios, and skill-station rotations. Next comes on-site observation of expert crews (3,148; 74.80%), followed by high-quality video demonstrations that can be paused and replayed (3,018; 71.71%). Case-based discussion forums attracted 2,754 (65.43%), expert professional guidance 2,628 (62.44%), and traditional thematic lectures 2,562 (60.87%).

Chi-square analysis across the five hospital strata shows no statistically significant difference in training method preferences overall (χ^2^ = 12.266, *P* = 0.907). This indicates that all staff, irrespective of workplace, recognize the primacy of repeated psychomotor rehearsal and practical skill acquisition. However, closer examination reveals that tertiary-grade-A hospitals showed higher absolute numbers for case discussion (1,392), on-site observation (1,564), and video-based teaching (1,424), suggesting that staff in these centers, who see the most complex cases, crave peer-to-peer deliberation and visual exemplars, whereas colleagues in lower-tier units may lack even the basic equipment to imitate what they observe (Table 6).

**TABLE 6:** Comparison of training-method preferences among pre-hospital emergency personnel by hospital level.

Training method	Hospital level					χ ^2^	*P*
	Tertiary Grade-A	Tertiary Grade-B	Secondary Grade-A	Secondary Grade-B	Below Secondary		
Case discussion	1392	443	245	171	503	12.266	0.907
On-site observation	1564	515	297	193	579		
Thematic lecture	1260	438	251	162	451		
Professional guidance	1288	449	256	162	473		
Video-based teaching	1424	549	304	194	547		
Practical operation	1742	619	331	220	656		

### 3.4.2 Content priorities differ by hospital level

The questionnaire listed six clinical domains and again permitted multiple choices. Environmental-injury management—heat stroke, hypothermia, altitude sickness, snake bite, lightning strike—heads the wish-list: 3,248 respondents (77.17%) asked for more content, a finding that reflects Chengdu’s mountainous surrounding counties and the city’s recent experience with floods and landslides. Trauma care comes second (3,087; 73.34%), cardiovascular emergencies third (2,822; 67.05%), toxicology fourth (2,787; 66.22%), stroke fifth (2,694; 64.01%), and disaster medicine sixth (2,787; 64.5%).

Cross-tabulation reveals that interest in cardiovascular, trauma, stroke, environmental-injury and disaster modules all vary significantly with hospital level (*P* < 0.05): grassroots crews, who are first on scene yet have the thinnest resource base, express the greatest urgency for trauma and disaster drills, whereas tertiary-grade-A staff, already proficient in advanced life support, prioritize fine-tuning in stroke and cardio-cerebrovascular pathways. Curiously, toxicology preference is uniform across all strata, probably because poisoning cases occur everywhere and follow similar decontamination and antidote algorithms regardless of hospital size. Taken together, these data argue for a modular curriculum in which core skills are standardized but satellite topics—environmental injury, disaster medicine, complex stroke—are offered as electives weighted toward the hospital levels that will actually use them (Table 7).

**TABLE 7:** Comparison of training-content preferences among pre-hospital emergency personnel by hospital level.

Training method	Hospital level					χ ^2^	*P*
	Tertiary Grade-A	Tertiary Grade-B	Secondary Grade-A	Secondary Grade-B	Below Secondary		
Cardiovascular emergency care	1,267	468	283	192	612	63.788	0.000
Trauma emergency care	1,466	511	311	215	584		
Stroke emergency care	1,182	459	286	193	574		
Environmental-injury emergency care	1,533	556	297	215	647		
Disaster medicine	1,400	434	237	155	458		
Poisoning emergency care	1,384	469	261	163	510		

## 4 Discussion

### 4.1 Profile of Chengdu’s pre-hospital workforce

Pre-hospital emergency personnel in Chengdu are mostly with bachelor’s degree or below (92.80%), mainly with working years between 0 and 10 years (52.08%), and predominantly with intermediate or below professional titles (92.14%). Compared with developed countries, emergency personnel in China have high work intensity, and in most areas, compensation is inversely proportional to risk, resulting in few people willing to engage in pre-hospital emergency work (9, 10). Hospitals are forced to lower employment standards for scarce specialties, so the educational level of pre-hospital emergency personnel is generally lower than that of specialized medical staff at the same level hospitals. China currently has no independent evaluation standards for professional title assessment of pre-hospital emergency personnel. Combined with factors such as work pressure and educational background, this has led to enormous difficulty in professional title promotion for pre-hospital emergency personnel.

### 4.2 Current training realities and experience-based needs

The channels for obtaining emergency medical knowledge among pre-hospital emergency personnel in Chengdu are relatively similar overall, with no significant difference by hospital level. However, important nuances emerge staff at tertiary-grade-A hospitals utilize academic resources and professional lectures more frequently, while grassroots personnel depend more on off-site and online training. This disparity in access to high-quality educational resources may contribute to the differing self-perceived competency gaps across experience levels.

Critically, our analysis reveals that weak areas vary significantly by years of work experience (χ^2^ = 203.797, *P* < 0.001). Novice staff (0–5 years) most commonly identify clinical experience and communication skills as deficiencies, reflecting their newcomer status and limited exposure to diverse emergency scenarios. Those with 5–10 years prioritize theoretical knowledge and operational skills, suggesting a consolidation phase where they seek to solidify their technical foundation. Mid-career personnel (10–15 years) show heightened concern about clinical thinking and department management, indicating growing administrative responsibilities and complex case exposure. Senior staff (>15 years) demonstrate more selective training needs, though operational skills remain a concern. Research capabilities are uniformly weak across all experience groups, ranging from 32.3 to 36.2%, underscoring a systemic neglect of research literacy that impedes professional advancement regardless of seniority (11–13).

With the implementation of the hierarchical diagnosis and treatment system, the workload of grassroots emergency personnel has increased, leading to fewer opportunities for external study and shorter study durations. Even when they participate in training, they are more likely to forget due to prolonged periods without using the skills. Research has found (11) that it may be reasonable for training units to develop appropriate emergency training plans and establish feasible retraining mechanisms, with retraining for personnel who received training at 3-month and 6-month intervals. Pre-hospital emergency personnel participate in training through continuing education methods such as full-time advanced studies and on-the-job academic degree advancement. Training departments can offer short-term training courses in fields such as emergency medicine, surgery, trauma treatment, and disaster response, which can also improve pre-hospital emergency personnel’s professional knowledge and skills regarding pre-hospital emergency system aspects such as emergency room construction, staffing, and equipment allocation (12). It is recommended that medical schools establish pre-hospital emergency specialties to cultivate pre-hospital emergency talents; hospitals should develop corresponding recruitment and incentive mechanisms to attract more medical students to join the pre-hospital emergency workforce. The low proportion of pre-hospital emergency personnel participating in research training may be related to pre-hospital emergency workload, pressure, as well as research thinking and emphasis, but research capability is to some extent associated with professional title promotion. Therefore, training departments should actively guide pre-hospital emergency personnel to participate in research. By establishing transparent salary systems and promotion mechanisms, the resignation rate of pre-hospital emergency personnel can be reduced, promoting the sustainable development of pre-hospital emergency medicine (13).

### 4.3 Deficits and remedies: the universal demand for practical skills

Practical operation skills are the most in need of improvement across all personnel. Pre-hospital patients typically have urgent and critical conditions with complex and rapidly changing disease states. Pre-hospital emergency personnel not only need to master common pre-hospital procedures such as basic life support, endotracheal intubation, and trauma management, but should also gradually master techniques such as intraosseous access, point-of-care ultrasound, and even extracorporeal cardiopulmonary resuscitation (ECPR) (14). Wang et al. (3) believe that with the extension of working time and the updating of emergency knowledge and skills, many medical personnel feel that their emergency capabilities are insufficient and outdated, and they desire guidance in standardized operational procedures to improve the proficiency of emergency skills.

Pre-hospital emergency personnel participating in training are mainly concerned about issues such as training content not being applicable, theory not matching practice, and uneven quality of training instructors, spending time without improving their emergency capabilities. Frenk et al. (15) believe that by constructing a competency model for pre-hospital emergency personnel to promote performance reform of the entire health system, personalized training content and methods can be developed to provide scientific and effective training for pre-hospital emergency personnel and improve their emergency response levels.

### 4.4 Rational development of pre-hospital emergency personnel training methods and content

#### 4.4.1 Training methods: universal preference for hands-on learning

Our findings reveal no statistically significant difference in training method preferences across hospital levels (*P* = 0.907), indicating that all pre-hospital emergency personnel—regardless of institutional tier—universally recognize the primacy of practical skill acquisition. Firsthand practice dominates preferences (84.77%), followed by on-site observation (74.80%) and video-based teaching (71.71%). This uniformity suggests that training infrastructure investments should prioritize simulation equipment, skill laboratories, and expert demonstration opportunities that serve all hospital levels equitably. While tertiary-grade-A hospitals show higher absolute participation in case discussions and video teaching, likely reflecting their greater resource availability—the fundamental preference structure remains consistent. This finding supports centralized training resource allocation with standardized simulation-based curricula, rather than divergent methodological approaches by hospital tier (16).

#### 4.4.2 Training content: stratified needs by hospital level

In contrast to method preferences, training content preferences differ significantly by hospital level (*P* < 0.05 for cardiovascular, trauma, stroke, environmental-injury, and disaster medicine; *P* = 0.095 for toxicology). The speculated reasons are as follows:

➀ Trauma and disasters usually occur in areas far from major cities. Grassroots pre-hospital emergency personnel are typically the first rescue force, but their patient volume and technical capabilities are limited, thus facing problems such as insufficient professional competence and low organizational management efficiency (17). Critically ill patients still require support from higher-level hospitals or transfer to higher-level hospitals, but providing basic pre-hospital trauma management, identifying severely traumatized patients, and timely taking necessary life-saving measures are very important for patient prognosis (18);
➁ The incidence and mortality rates of cardiovascular and cerebrovascular diseases are significantly higher in rural areas than in cities. The key to treatment lies in identifying and stabilizing the condition at the first moment. The main task of pre-hospital emergency personnel is to identify such diseases, then allow patients to receive professional treatment at corresponding hospitals through models such as chest pain centers and stroke centers (19, 20);
➂ Consistent with foreign research (21), poisoning incidents occur in all regions, but due to differences in geography, population base, and economic development, the timing and types of poisoning vary, and everyone’s needs differ—explaining the uniform preference for toxicology training.
➃ Different pre-hospital emergency personnel are responsible for different stages in the disease diagnosis and treatment process; therefore, their choices of training content differ. The prominent demand of pre-hospital emergency personnel for environmental injury emergency training may be related to regional characteristics such as complex geographical environments and frequent extreme weather events, which can be emphasized in training design.

The United States divides pre-hospital emergency personnel into 4 categories, with different levels of emergency personnel required to master different skills: first responders need to master simple first aid techniques; EMT-Basics need to master basic life support techniques and on-site trauma first aid techniques; EMT-Intermediates need to master basic life support techniques, basic on-site trauma first aid techniques, and endotracheal intubation; EMT-Paramedics need to master on-site trauma first aid techniques and advanced life support techniques (5). Chengdu can learn from American experience, combined with survey results, to develop different training contents and methods for different level hospitals.

#### 4.4.3 Experience-stratified training: a missing dimension

Given the significant variation in self-identified weak areas by work experience, training programs should incorporate vertical stratification by career stage alongside horizontal stratification by hospital level. Novice personnel require mentorship and communication skills development; mid-career staff need advanced clinical reasoning and management training; senior practitioners benefit from specialized technical updates and research methodology instruction. This dual-stratification approach—by both institutional level and professional experience—would address the full spectrum of competency gaps identified (22–25).

Li et al. (22) used a first aid training program combining theory + video + scenario models, allowing trainees to actively, proactively, and repeatedly practice through role-playing, equipment use, practical operations, and team cooperation in targeted simulation environments, not only consolidating individual skills but also improving team tacit understanding, thereby enhancing emergency response capabilities. Lu et al. (23) used an immersive virtual reality training system that allowed trainees to directly operate various surgical instruments while watching stereoscopic three-dimensional surgical videos, enabling trainees to simulate real battlefield environments immersively, thereby improving training effectiveness. Research has found (24) that the Entrustable Professional Activities training program can effectively improve the decision-making ability of medical students with different training years, different educational backgrounds, and different specialties, while also improving their ability to continuously manage patients to a certain extent. Multiple real clinical scenarios are constructed to form a systematic and complete Entrustable Professional Activities training program, improving the core competency capabilities such as theory and practice of trainees (25).

Thus, it can be seen that different regions, different hospital business capabilities, different professional experience levels, and different regional disease incidence rates lead to different training needs for pre-hospital emergency personnel. Training units should develop appropriate training contents and methods according to actual needs (26, 27). Chengdu currently conducts BLS training for all pre-hospital emergency personnel to consolidate basic skills such as cardiopulmonary resuscitation and AED. However, according to the results of this survey, pre-hospital emergency personnel at different level hospitals and different career stages should be stratified for training, and practice-oriented, highly practical training should be carried out.

Pre-hospital emergency care is the cornerstone of reducing disability and mortality rates and has become a focus of attention in the medical community and even among the entire population. Developing standardized and efficient emergency training plans and assessment standards, improving the emergency capabilities of pre-hospital emergency personnel, and promoting the sustainable development of emergency medicine are long-term and arduous tasks. This survey provides a scientific basis for developing a sound pre-hospital emergency training system in Chengdu.

## 5 Conclusion

Pre-hospital emergency care is the cornerstone for reducing disability and mortality and has become a focus of both the medical profession and the public. Formulating standardized, efficient training plans and assessment criteria to improve pre-hospital capability and promote sustainable development of emergency medicine remains a long and arduous task. The present survey provides a scientific basis for establishing a sound pre-hospital training system.

## Data Availability

The raw data supporting the conclusions of this article will be made available by the authors, without undue reservation.
